# Antiretroviral therapy timing impacts latent tuberculosis infection reactivation in a *Mycobacterium tuberculosis*/SIV coinfection model 

**DOI:** 10.1172/JCI153090

**Published:** 2022-02-01

**Authors:** Riti Sharan, Shashank R. Ganatra, Allison N. Bucsan, Journey Cole, Dhiraj K. Singh, Xavier Alvarez, Maya Gough, Cynthia Alvarez, Alyssa Blakley, Justin Ferdin, Rajesh Thippeshappa, Bindu Singh, Ruby Escobedo, Vinay Shivanna, Edward J. Dick, Shannan Hall-Ursone, Shabaana A. Khader, Smriti Mehra, Jyothi Rengarajan, Deepak Kaushal

**Affiliations:** 1 Southwest National Primate Research Center, Texas Biomedical Research Institute, San Antonio, Texas, USA.; 2 Department of Molecular Microbiology, Washington University in St. Louis School of Medicine, St. Louis, Missouri, USA.; 3 Tulane National Primate Research Center, Tulane University School of Medicine, Covington, Louisiana, USA.; 4 Emory Vaccine Center and Yerkes National Primate Research Center, Emory University School of Medicine, Atlanta, Georgia, USA.

**Keywords:** AIDS/HIV, Infectious disease, Tuberculosis

## Abstract

Studies using the nonhuman primate model of *Mycobacterium* *tuberculosis*/simian immunodeficiency virus coinfection have revealed protective CD4^+^ T cell–independent immune responses that suppress latent tuberculosis infection (LTBI) reactivation. In particular, chronic immune activation rather than the mere depletion of CD4^+^ T cells correlates with reactivation due to SIV coinfection. Here, we administered combinatorial antiretroviral therapy (cART) 2 weeks after SIV coinfection to study whether restoration of CD4^+^ T cell immunity occurred more broadly, and whether this prevented reactivation of LTBI compared to cART initiated 4 weeks after SIV. Earlier initiation of cART enhanced survival, led to better control of viral replication, and reduced immune activation in the periphery and lung vasculature, thereby reducing the rate of SIV-induced reactivation. We observed robust CD8^+^ T effector memory responses and significantly reduced macrophage turnover in the lung tissue. However, skewed CD4^+^ T effector memory responses persisted and new TB lesions formed after SIV coinfection. Thus, reactivation of LTBI is governed by very early events of SIV infection. Timing of cART is critical in mitigating chronic immune activation. The potential novelty of these findings mainly relates to the development of a robust animal model of human *M*. *tuberculosis*/HIV coinfection that allows the testing of underlying mechanisms.

## Graphical abstract

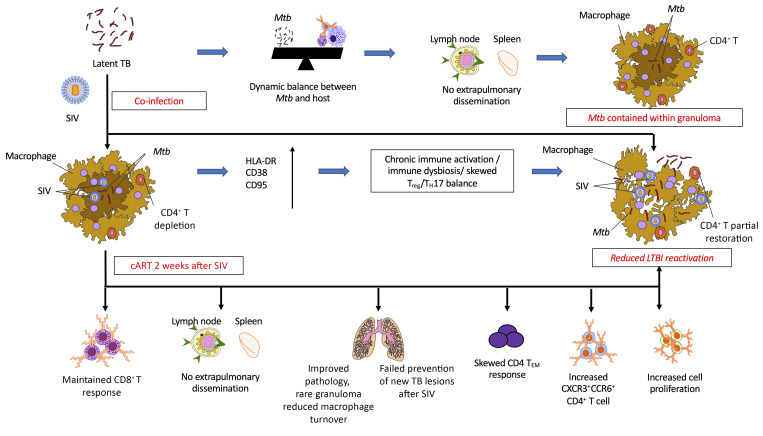

## Introduction

The tuberculosis and HIV copandemic continues to pose a major healthcare burden in resource-limited countries (1). HIV coinfection predisposes the host to reactivation of latent tuberculosis infection (LTBI), resulting in worsening of disease conditions, and in mortality. Epidemiological studies in the United States estimate that HIV-infected persons have a 9-times higher chance of progressing to active TB from LTBI compared with HIV-uninfected individuals (2). Approximately 0.08% to 2% of people with LTBI experience reactivation following HIV coinfection and combinatorial antiretroviral therapy (cART) treatment (3, 4). The reason that some reactivate while others do not is not completely understood, with the sequence of *Mycobacterium* *tuberculosis* (*Mtb*) and HIV infections playing a pivotal role in humans. The best-characterized impact of HIV on immune function is the depletion of CD4^+^ T cells in lymphoid tissues and peripheral blood (5, 6). Additionally, the frequency and depletion of CD4^+^ T cells in lungs and lymph nodes is a strong predictor of progression to TB. Studies using the nonhuman primate (NHP) model of *Mtb*/simian immunodeficiency virus (SIV) (7) coinfection have revealed that while CD4^+^ T cell–dependent mechanisms are important in LTBI reactivation in an SIV coinfection setting (8), there are CD4^+^ T cell–independent immune responses that suppress and therefore protect against LTBI reactivation (9, 10). However, antibody-mediated depletion of CD4^+^ T cells in *Mtb*/SIV-coinfection studies has revealed that the virus-mediated TB-specific disruption of immune response goes beyond CD4^+^ T cell depletion (11). These studies suggest a role of granuloma-specific responses as more critical in LTBI reactivation than just peripheral CD4^+^ cell counts (12). We have previously shown that there are direct cytopathic effects of SIV resulting in chronic immune activation, altered effector T cell phenotypes, and dysregulated T cell homeostasis that causes LTBI reactivation (10, 13). Further, highly effective cART (14), while effective in reducing viral loads in the periphery and lungs of *Mtb*/SIV-coinfected macaques, failed to reduce the rate of reactivation of LTBI (15). Thus, it is important to understand the driving forces behind chronic immune activation in a relevant coinfected preclinical model. This will lead to the discovery of key biomarkers predicting LTBI reactivation and therefore point the way to intervention strategies (13, 16).

We aim to leverage our NHP model of *Mtb*/SIV coinfection to study which concurrent cART/anti-TB regimens are most effective in controlling the chronic immune activation and subsequent LTBI reactivation. Previously, a greater effect of combined cART and isoniazid preventive therapy (IPT) on reducing the risk of TB has been shown compared with cART alone (17–19). The TEMPRANO ANRS clinical trial demonstrated that a 6-month IPT and early cART each independently reduced mortality in HIV-infected people in Cote d’Ivoire with high CD4^+^ cell counts (20). Additionally, IPT with early cART provided marked protection from a serious HIV-related event or death. Despite IPT being the cornerstone of protection against TB in HIV^+^ patients during cART, it is plausible that protection is lost after ending therapy (21). We hypothesized that it is imperative to optimize the timing of antiviral therapy to effectively control immune activation before initiating antibacterial therapy. The optimal time to initiate cART in adults who are infected with HIV is as early as possible based on data from several randomized clinical trials in humans (22–26). The general practice to defer initiation of cART in HIV^+^ asymptomatic individuals has changed over time (27). The Strategic Timing of Antiretroviral Therapy (START) study concluded that there was a substantial benefit in immediate initiation of cART in HIV^+^ patients irrespective of CD4^+^ cell count (22). In addition, the Starting Antiretroviral Therapy at Three Points in Tuberculosis (SAPIT) trial provided compelling evidence of the benefit of initiating cART during anti-TB therapy in HIV-coinfected patients (24). However, it has been noted that the rapidity of the restoration of *Mtb* responses upon cART is fairly rapid (28, 29). This is evidenced by the fact that TB patients who develop immune reconstitution disease typically do so during the first few weeks after the initiation of cART (30). However, long-term recovery of TB-specific immune function is incomplete (31). To study the impact of the timing of cART on the immune activation, we utilized our established cART-treated *Mtb*/SIV-coinfected rhesus macaque model.

The aim of this study was to determine whether administering cART at peak viremia (2 weeks after SIV coinfection) compared with cART at chronic phase of the virus (4 weeks after SIV coinfection) is able to rescue from the virus-induced immune activation and prevent LTBI reactivation. Though our *Mtb* infection mimics the human route and is likely only 1-log different than the human dose (we infect with ~10 CFU via aerosol), we do not infect using either the natural route or the physiologically relevant dose of HIV (we infect with 300 TCID_50_ [50% tissue culture infective dose] SIVmac_239_ via i.v. route) in our model. This approach is necessary to reduce both the time and number of animals in these experiments while appropriately powering them. In the present study, we identified an immune correlate of LTBI reactivation, namely, macrophage turnover, in macaque lungs.

Although initiating cART earlier leads to better survival in humans, we show for the first time to our knowledge that initiating cART earlier results in reduced macrophage turnover in the lungs of coinfected rhesus macaques. The updated guidelines for use of cART in *Mtb*/HIV-coinfected individuals recommends the use of once-daily dolutegravir (DTG) or twice-daily raltegravir (400 mg daily) in combination with tenofovir disoproxil fumarate/emtricitabine with once-weekly isoniazid plus rifapentine for 3 months (32). Most highly active ART (HAART) treatment regimens for humans include drugs from at least 2 of the 3 classes of cART (nucleoside analog reverse transcriptase [RT] inhibitors, non-nucleoside analog RT inhibitors, and protease inhibitors; ref. 33). In accordance with the recommendations, we utilized an established cART regimen consisting of a formulation of a 3-drug cocktail containing RT inhibitors tenofovir (20 mg/kg), emtricitabine (30 mg/kg), and the integrase inhibitor DTG (2.5 mg/kg) (34–39). To determine whether the early timing of cART enhanced survival and/or had an impact on the bacterial control, we compared the data from the current study (cART administered 2 weeks after SIV coinfection) with the published data from coinfected macaques that were administered cART 4 weeks after SIV coinfection (15). We aimed to identify the components of TB immunity in the blood and lung compartments that remain impaired after cART, versus those that are restored by cART administered 2 weeks after SIV coinfection (cART/2 week) and at 4 weeks after SIV coinfection (cART/4 week) in our model.

We were able to demonstrate that cART/2 week enhanced the general well being of the study macaques, as evidenced by an increased survival during the protocol. cART/2 week controlled the viral replication and significantly reduced immune activation in BAL and blood, thereby reducing the rate of SIV-driven LTBI reactivation. This was consistent with improved lung pathology in this group compared with the cART-naive or the cART/4-week group. Computed tomography (CT) imaging of the lungs demonstrated a severe bronchial lymphadenopathy upon cART administration at 4 weeks after SIV. Despite lowering inflammation and pathology due to an earlier and effective control of SIV infection, cART/2 week failed to reconstitute the skewed effector memory responses in the lung compartment. Depleted CD4^+^ T cells were only partially restored and a marked increase in Th1 responses was still observed in the cART/2-week group. A higher percentage of CXCR3^+^CD4^+^ and CCR6^+^CD4^+^ T cells was observed in cART/2-week group in both BAL and whole blood at the end of the study. Further studies aiming at (a) concurrent therapies to contain bacterial burden and (b) to study the impact of early initiation of cART to maintain the gut integrity and reduce microbial translocation are needed to have an optimal translational intervention.

## Results

### cART at peak viremia (cART/2 week) increases survival.

**Figure 1:**
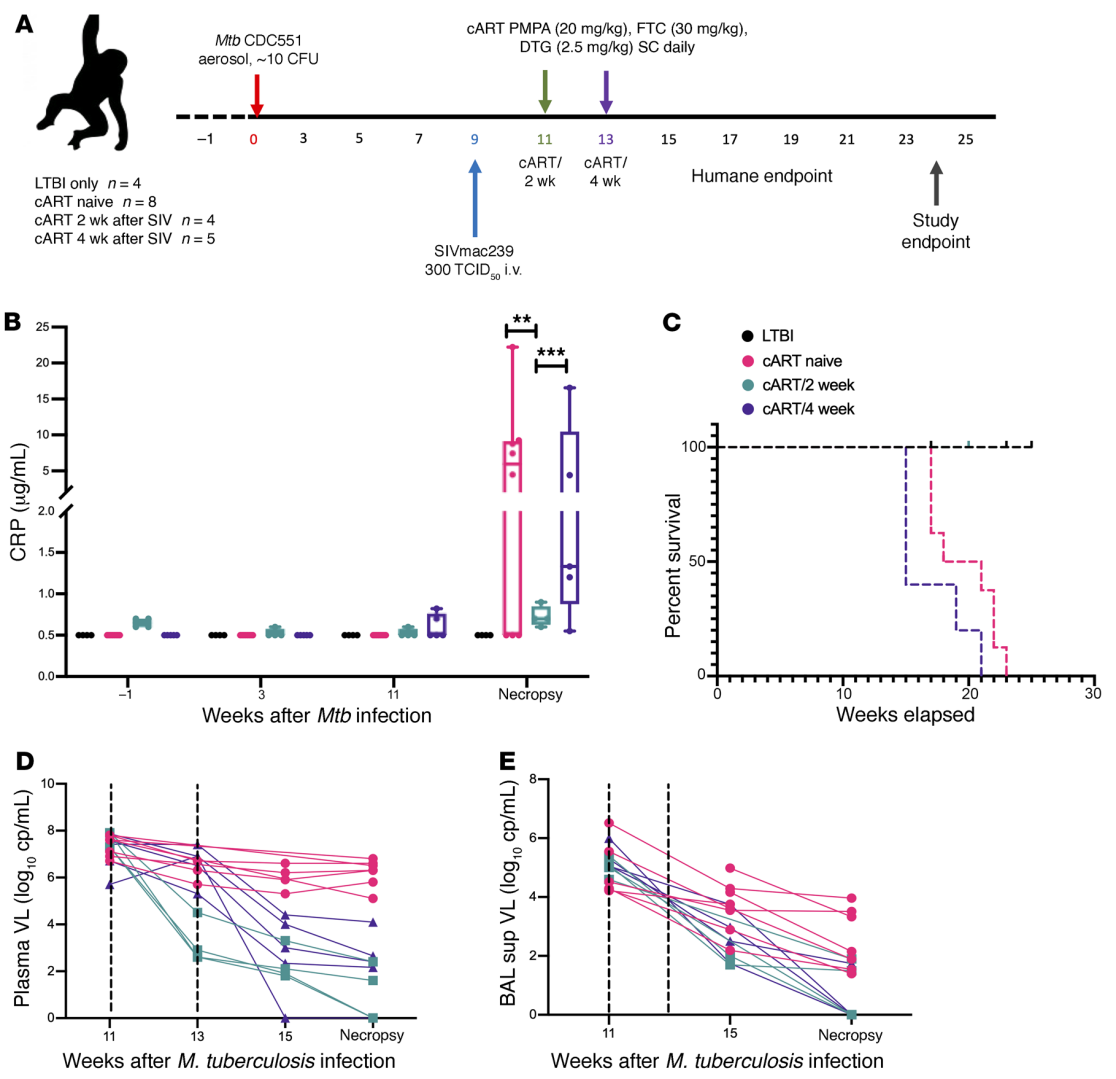
Comparison of clinical parameters in LTBI, cART naive, cART/2 week, and cART/4 week. (**A**) Study outline. (**B**) Serum CRP levels. (**C**) Survival curve representing the 4 study groups: LTBI (*n* = 4), cART naive (*n* = 8), cART/2 week (*n* = 4), and cART/4 week (*n* = 5). The survival curves were compared using the log-rank, Mantel-Cox, or Gehan-Breslow-Wilcoxon test. Viral loads in (**D**) plasma and (**E**) BAL supernatants of the treated macaques were measured longitudinally throughout the study. Data in **B**, **D**, and **E** were analyzed using 1-way ANOVA with Tukey’s multiple-comparison test. **P < 0.01, ***P < 0.001. Data are presented as mean ± SEM.

To assess the impact of differential timing of cART on LTBI reactivation, we utilized 5 new macaques (cART/2 week, *n* = 4; cART/4 week, *n* = 1) infected with a low dose of approximately 10 CFU *Mtb* CDC1551 and reused published data from LTBI (*n* =4), cART-naive coinfected macaques (*n* = 8), and cART/4 week (*n* = 4) (ref. 15 and Supplemental Table 1; supplemental material available online with this article; https://doi.org/10.1172/JCI153090DS1). The study design is outlined in Figure 1A. All the macaques were infected with a low dose of *Mtb* (~10 CFU deposited in the lungs) and SIV (300 TCID_50_ SIVmac_239_). Infection was confirmed by a positive tuberculin skin test (40) at weeks 3 and 5 after *Mtb* infection. All macaques in the study developed asymptomatic LTBI infection characterized by less than 1 to 2 log_10_CFU of *Mtb* in the BAL at weeks 3, 5, and 7 after *Mtb* infection, serum C-reactive protein (CRP) of 5 μg/mL or lower (Figure 1B), and no significant difference in percentage body temperature (Supplemental Figure 1A) and body weight (Supplemental Figure 1B) up to 9 weeks after *Mtb* infection. Upon establishment of latency, macaques were coinfected with 300 TCID_50_ SIVmac_239_ via the intravenous route 9 weeks after *Mtb* infection (9, 10, 15). Once confirmation of SIV infection was evidenced by plasma viral loads measured via reverse transcriptase quantitative PCR (RT-qPCR), the macaques were treated with cART. The clinical, pathological, and immunological responses were studied in the 4 experimental groups: LTBI, cART naive, cART/2 week, and cART/4 week.

Survival was a critical correlate impacted by the timing of cART in this study. There was a significant difference in the survival curves of the 4 experimental groups (*P* = 0.0006; Mantel-Cox test). Macaques in group cART/2 week survived in good body condition with adequate body muscling and fat until the predetermined study endpoint (Figure 1C), significantly longer than macaques in group cART/4 week (*P* = 0.02). Conversely, macaques in group cART/4 week were humanely euthanized based on prespecified endpoints starting as early as 1 week after cART initiation (Figure 1C). The macaques in group cART/2 week survived longer than cART naive and macaques in group cART/4 week survived for a reduced period compared with cART naive. These differences were not significant as determined by Mantel-Cox and Gehan-Breslow-Wilcoxon tests. The clinical signs of active TB in humans and NHPs is often associated with elevated serum CRP levels, declining body weight, and increased body temperature (41, 42). CRP is an inflammatory marker of disease severity that correlates with bacterial burden in NHPs (9, 42). CRP levels were significantly lower in the macaques in group cART/2 week at study endpoint compared with the macaques from the cART/4-week group (*P* < 0.001) and cART-naive controls (*P* < 0.0001) (Figure 1B). It is to be noted that the low CRP levels in the LTBI group are expected due to the low bacterial burden (42). The macaques in group cART/2 week maintained low CRP values, with not more than 5%–7% body weight loss or fever (Figure 1B).

### cART effectively controls viral replication.

To evaluate the efficacy of the cART regimen, viral loads were measured in the plasma and BAL supernatant of all the coinfected and treated macaques. No significant difference in plasma and BAL supernatant viral loads was observed in the cART-naive coinfected macaques between the peak viremia (week 11 after *Mtb* or 2 weeks after SIV) and study endpoint (Figure 1, D and E). A significant and rapid decrease in the viral loads of plasma and BAL supernatant was observed at necropsy compared with peak of viremia in both the cART/2-week and cART/4-week groups (~4 log, *P* < 0.0001; Figure 1, D and E).

### Reduced bacterial burden with no extrapulmonary spread of Mtb upon earlier cART initiation.

**Figure 2:**
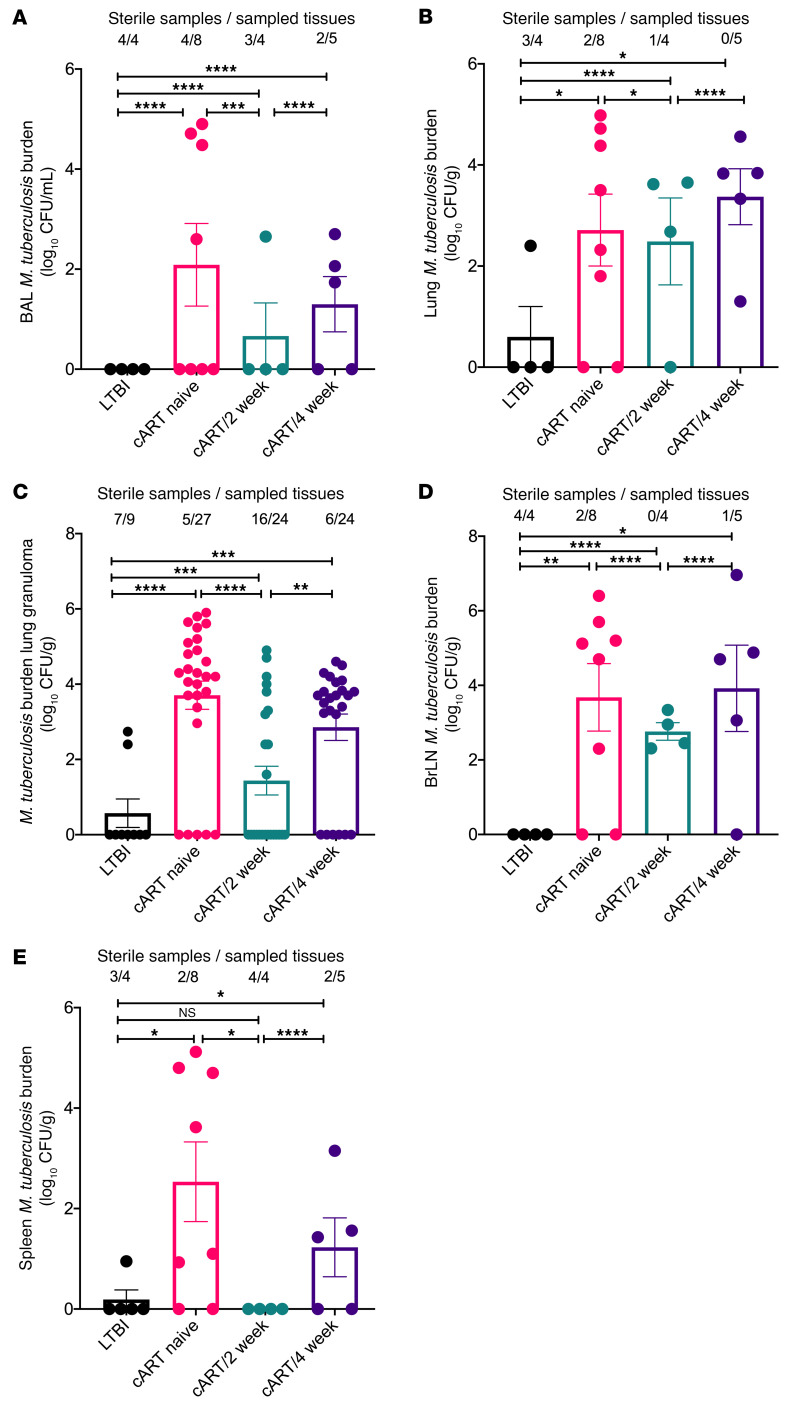
Impact of cART treatment on *Mtb* burden in tissues at necropsy. Bacterial burden (log_10_ CFU/mL or log_10_ CFU/g) was determined in the (**A**) BAL, (**B**) lungs, (**C**) lung granulomas, (**D**) bronchial lymph nodes (BrLN), and (**E**) spleen at necropsy by homogenizing the tissues and plating on agar plates. Significance was determined in LTBI (*n* = 4), cART naive (*n* = 8), cART/2 week (*n* = 4), and cART/4 week (*n* = 5) using 1-way ANOVA with Tukey’s multiple-comparison test. *P < 0.05; **P < 0.01; ***P < 0.001; ****P < 0.0001. Data are presented as mean ± SEM.

To determine the impact of cART timing on bacterial burden, BAL fluid, lungs, spleens, bronchial lymph nodes, and lung granulomas were plated on agar plates as described previously (9, 43). The macaques in group cART/2 week had significantly lower bacterial burden (*P* = 0.0003, <1 × 10^2^ CFU/g in 3 out of 4 macaques sampled) compared with the cART-naive and cART/4-week groups at necropsy (Figure 2A). cART-naive macaques (*P* = 0.0182, ~1 × 10^4^ CFU/g) and macaques in group cART/4 week displayed a significantly higher burden (*P* = 0.0002, ~1 × 10^3^ CFU/g) in the lung tissue when compared with LTBI controls and the cART/2-week group (Figure 2B). The bacterial burden was significantly higher in the lung granulomas of macaques in the cART-naive (*P* < 0.0001), cART/2-week (*P* = 0.001), and cART/4-week (*P* = 0.0009) groups compared with LTBI controls (Figure 2C). However, the burden in the lung granulomas of macaques in group cART/2 week was significantly (*P* = 0.0087) lower than in group cART/4 week (Figure 2C). Similar to lungs and lung granulomas, a significantly higher bacterial burden was observed in the bronchial lymph nodes of macaques in the cART-naive (*P* = 0.0053), cART/2-week (*P* < 0.0001), and cART/4-week (*P* = 0.02) groups compared with the LTBI controls (Figure 2D). The bacterial burden was significantly reduced (*P* < 0.0001) in bronchial lymph nodes of macaques in group cART/2 week compared with the macaques in groups cART naive and cART/4 week (Figure 2D). No extrapulmonary spread of bacteria was observed in the spleen of all 4 macaques in group cART/2 week (Figure 2E). In contrast, 3 out of 5 macaques in group cART/4 week displayed approximately 1 × 10^2^ CFU/g in the spleen at necropsy (Figure 2E).

### Reduced granuloma formation and improved lung pathology upon earlier cART initiation.

**Figure 3:**
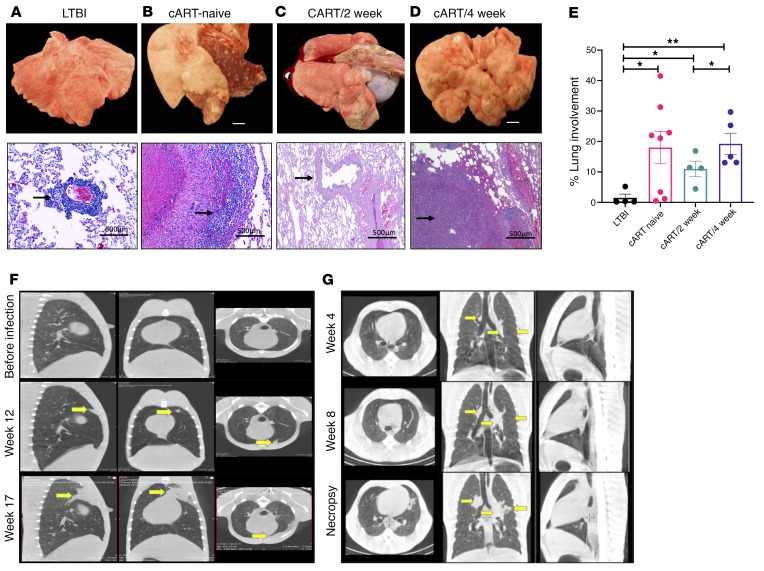
Impact of timing of cART on lung pathology and TB lesions in *Mtb*/SIV-coinfected macaques. To determine the impact of cART timing on lung pathology, lung tissue was collected at necropsy (top images) and stained with H&E (bottom images) to study the cellular and granulomatous pathology in (**A**) LTBI (*n* = 4), (**B**) cART naive (*n* = 8), (**C**) cART/2 week (*n* = 4), and (**D**) cART/4 week (*n* = 5). Scale bars: 500 μm. (**E**) Percentage lung involvement was calculated by a board certified pathologist by quantification of the number of lesions per lung lobe. (**F**) Computed tomography (CT) imaging was performed on the macaques that received cART 2 weeks after SIV coinfection at different time points throughout the study to examine the TB lesions before SIV, after SIV, and after cART. (**G**) CT imaging was performed on the macaques that received cART 4 weeks after SIV coinfection at different time points throughout the study to examine the TB lesions before SIV, after SIV, and after cART. Black arrows in **A**–**D** indicate inflammation associated with granulomatous region. Yellow marks in **F** and **G** indicate the worsening of lung lesions. Significance was determined using 1-way ANOVA with Tukey’s multiple-comparison test. *P < 0.05; **P < 0.01. Data are presented as mean ± SEM.

To determine the impact of timing of cART on the lung pathology, lung tissue was collected at necropsy and stained with H&E to study the cellular and granulomatous pathology (Figure 3). The pathological findings correlated well with the clinical and microbiological findings. The LTBI group expectedly had few to no granulomas, with an average of 4% to 5% lung involvement (Figure 3, A and E). This group also displayed reduced TB disease–related pathology, including edema, pneumonia, and generalized foci of inflammation (Figure 3A). In contrast, the coinfected cART-naive group demonstrated a significantly higher (*P* < 0.05) lung involvement than the LTBI control group (Figure 3, B and E). This group exhibited lesions consistent with SIV-induced pathology, including interstitial pneumonia and septal thickening, increased accumulation of foamy alveolar macrophages, and lymphangitis (Figure 3B). The macaques in group cART/2 week demonstrated rare small granulomas (Figure 3, C and E) and minimal enlargement of hilar and bronchial lymph nodes (data not shown). Overall, the macaques in this group had good body condition, with fewer granulomas and less disease pathology (Supplemental Figure 1C). Gross pathology demonstrated that the macaques in group cART/4 week harbored numerous large granulomas, with significantly higher (*P* = 0.004) percentage lung involvement compared with LTBI controls (Figure 3, D and E). H&E staining demonstrated confluent granulomas with necrotic cores in both the cART-naive and cART/4-week groups (Figure 3, B and D). Overall, earlier initiation of cART resulted in significantly (*P* = 0.03) reduced lung involvement in group cART/2 week compared with group cART/4 week (Figure 3E).

### Earlier cART initiation does not prevent the formation of new TB lesions after SIV infection.

Computed tomography (CT) imaging was performed on the macaques in group cART/2 week at different time points throughout the study to examine TB lesions before SIV, after SIV, and after cART (Figure 3F). The findings were compared to the CT images of the macaque in group cART/4 week (Figure 3G). CT helped identify the lesions, TB reactivation, and the granulomatous regions of the lungs. CT scans demonstrated that the worsening of the pathology was significantly mitigated in group cART/2 week; earlier cART initiation was unable to rescue from new TB lesions (Figure 3F; weeks 12 and 17 after *Mtb*; Supplemental Figure 1D). The lung lesions in *Mtb* infection were characterized by solitary focal soft tissue attenuating nodules, usually subcentimeter in size (Figure 3G; week 4 after *Mtb*). In the longitudinal scans studied, the TB lung lesions did not show progression in numbers or size. During 2 to 3 weeks after intravenous SIV challenge (Figure 3F, week 12; Supplemental Figure 1D, weeks 8 and 12 after *Mtb*), an increase in the size of the preexisting nodules along with additional pulmonary nodules across several lung lobes was observed. As the disease progressed after SIV challenge, numerous, large, and irregular nodules extending into the lung periphery and the pleural margins were observed in the macaque in group cART/4 week (Figure 3G; week 8 and necropsy). Macaques in group cART/4 week demonstrated clinical signs of TB reactivation and developed severe alveolar pulmonary patterns with adjacent nodules, while some large nodular masses ranging from 1 to 1.5 cm in size were also recorded (Figure 3G; week 8 and necropsy). Additionally, the macaques in group cART/4 week (Figure 3G) demonstrated a severe disease progression in the lymph nodes at necropsy (1 week of cART). This demonstrated a severe lymphadenopathy, highlighting the possibility of a paradoxical reaction to TB after cART named immune reconstitution inflammatory syndrome (TB-IRIS) (Figure 3G). However, future studies with additional markers are needed to verify this occurrence of an IRIS-like phenotype in coinfected and cART-treated macaques.

### Earlier initiation of cART fails to restore CD4^+^ effector memory T cell responses.

**Figure 4:**
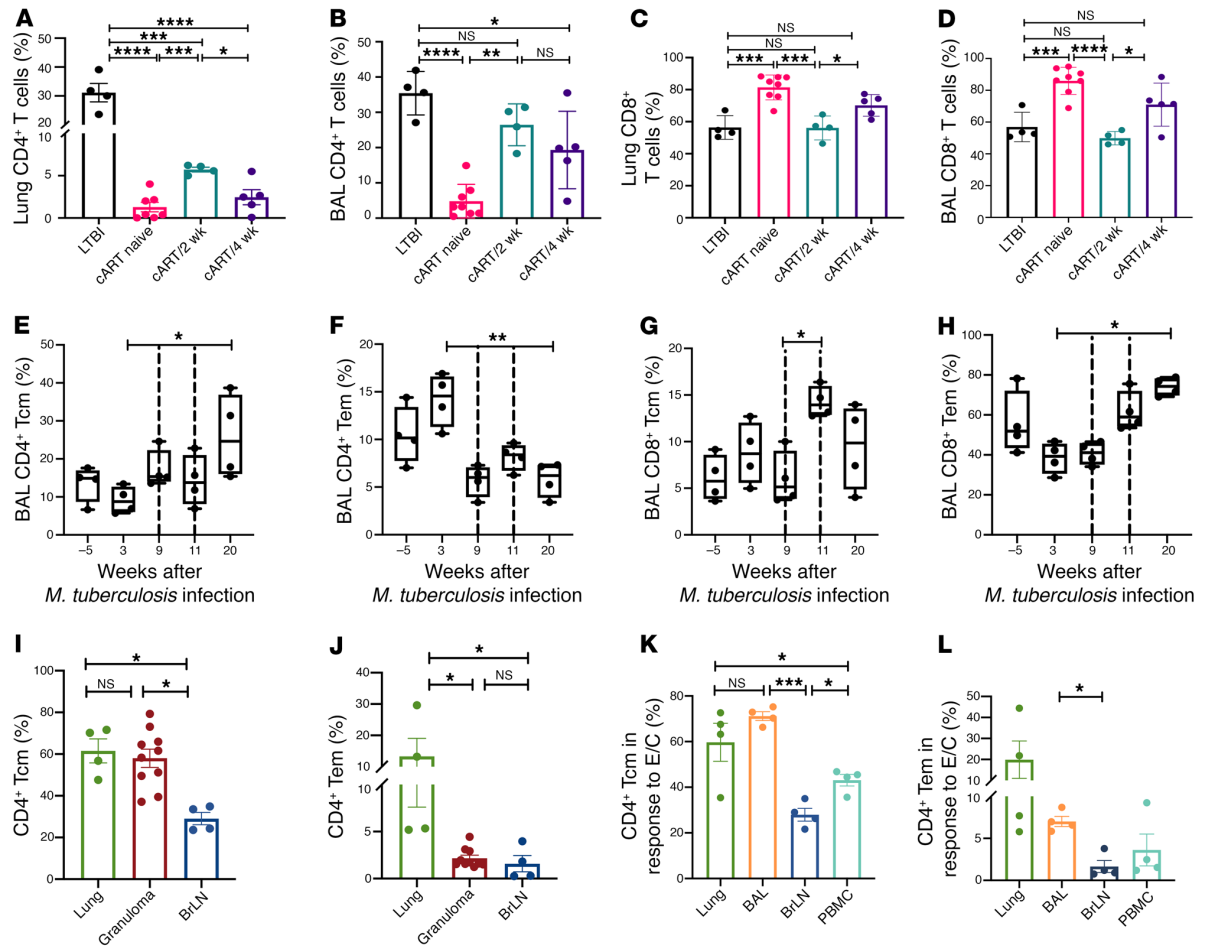
Earlier initiation of cART fails to restore CD4^+^ effector memory responses. To assess the impact of cART treatment on CD4^+^ T cell restoration, cells were stained with flow cytometry surface antibodies and acquired on a BD FACSymphony. Percentages of CD4^+^ T cells in (**A**) lungs and (**B**) BAL, and percentages of CD8^+^ T cells in (**C**) lungs and (**D**) BAL were determined in LTBI (*n* = 4), cART naive (*n* = 8), cART/2 week (*n* = 4), and cART/4 week (*n* = 5). Phenotyping of (**E**) BAL CD4^+^ Tcm cells and (**F**) CD4^+^ Tem was performed by staining for CD28^+^CD95^+^ (Tcm) and CD28^–^CD95^+^ (Tem) in cART/2 week (*n* = 4). Percentage of BAL (**G**) CD8^+^ Tcm and (**H**) CD8^+^ Tem cART/2 week (*n* = 4). (**I**) Percentages of CD4^+^ Tcm and (**J**) CD4^+^ Tem in lungs, granulomas, and bronchial lymph nodes (BrLN) of cART/2 week (*n* = 4). (**K**) Percentages of *Mtb*-specific CD4^+^ Tcm and (**L**) CD4^+^ Tem in lung, BAL, BrLN, and PBMCs of macaques in the cART/2-week group (*n* = 4) at necropsy. E/C refers to *Mtb* ESAT-6/CFP-10 antigen peptide. Significance was determined using 1-way ANOVA with Sidak’s or Tukey’s correction as applicable. *P < 0.05; **P < 0.01; ***P < 0.001; ****P < 0.0001. Data are presented as mean ± SEM.

cART treatment resulted in partial restoration of CD4^+^ T cells in the lung tissue (Figure 4A). Despite treatment, the percentage of CD4^+^ T cells remained significantly lower than in the LTBI control group (*P* < 0.0001). However, earlier initiation of cART resulted in a significantly higher percentage of CD4^+^ T cells in the lung tissue (Figure 4A) and BAL (Figure 4B) compared with both cART-naive (*P* = 0.003) and cART/4-week groups (*P* = 0.01). Additionally, there was a significant difference in both lung (*P* = 0.02) and BAL CD8^+^ T cells (*P* = 0.02) between the cART/2-week and cART/4-week groups (Figure 4, C and D). No significant impact of the timing of cART was observed on the CD4^+^ and CD8^+^ T cell responses in whole blood (Supplemental Figure 2, A and B), bronchial lymph nodes (Supplemental Figure 2, C and D), and spleen (Supplemental Figure 2, E and F). Further longitudinal phenotyping of the replenished BAL CD4^+^ T cells in the cART/2-week group demonstrated a higher percentage of central memory phenotype (Figure 4E). However, earlier initiation of cART could not rescue from the skewed CD4^+^ T effector memory response as evidenced by the significantly lower percentage of this subset at study endpoint (*P* = 0.017) compared with the pre-SIV levels (Figure 4F). There was no significant impact of early timing of cART initiation on CD8^+^ T central memory response, with the levels being maintained at similar percentages throughout the study period (Figure 4G). In contrast to CD4^+^ T cells, initiating cART 2 weeks after SIV significantly increased the CD8^+^ T effector memory response (Figure 4H) compared with the LTBI phase. There was no significant impact of initiating cART/2 week on peripheral CD4^+^ T central and effector memory responses (Supplemental Figure 3, A and B). Though early initiation of cART resulted in a significant increase in CD8^+^ T central memory response in the periphery (Supplemental Figure 3C), there were no significant changes in CD8^+^ T effector memory response (Supplemental Figure 3D). In conclusion, earlier initiation of cART is unable to restore the CD4^+^ T effector memory response in the BAL to levels maintained during the LTBI phase, possibly leading to reactivation despite adequate CD8^+^ T cell responses.

Further analysis of the restoration of CD4^+^ and CD8^+^ T cells in granulomas of the 2 treatment groups demonstrated a significantly higher (*P* < 0.0001) restoration of CD4^+^ T cells (Supplemental Figure 3E) and a lower percentage of CD8^+^ T cells (Supplemental Figure 3F) in the cART/2-week compared with the cART/4-week group. Since we observed a failure of restoration of adequate effector memory phenotype in the BAL with earlier cART initiation, we analyzed the levels of memory phenotype in different compartments of macaques in the cART/2-week group. A significantly lower percentage (*P* < 0.05) of both CD4^+^ T central (Figure 4I) and effector (Figure 4J) memory responses was observed in the bronchial lymph nodes compared with the lung compartment. No significant difference was observed in the CD8^+^ T central memory response in different compartments but there were significant differences in the CD8^+^ T effector memory responses (Supplemental Figure 3, G and H). Further studies aimed at studying the restoration of these responses in an anti-TB plus cART treatment model would better define the critical role of efficient effector memory responses in controlling LTBI reactivation.

Next, *Mtb*-specific CD4^+^ T central and effector memory responses in the lungs, BAL, bronchial lymph nodes, and PBMCs of macaques initiated on cART/2 week were analyzed. Approximately 60% *Mtb*-specific CD4^+^ T central (Figure 4K) and approximately 15% CD4^+^ T effector (Figure 4L) memory cells were observed in the lungs and BAL of macaques in the cART/2-week group. While no significant differences were observed in the *Mtb*-specific CD8^+^ T central memory responses (Supplemental Figure 3I), there were significantly lower percentages of CD8^+^ T effector memory cells in BAL, bronchial lymph nodes, and the periphery compared with the lung tissue (Supplemental Figure 3J). No significant difference was observed in the IFN-γ–producing *Mtb*-specific cells at necropsy in the different compartments (data not shown). We aim to perform a comparison of the *Mtb*-specific responses between LTBI, cART naive, cART/2 week, and cART/4 week in our future studies to correlate these findings with the bacterial load and LTBI reactivation.

### cART initiated at peak viremia better controls immune activation.

**Figure 5:**
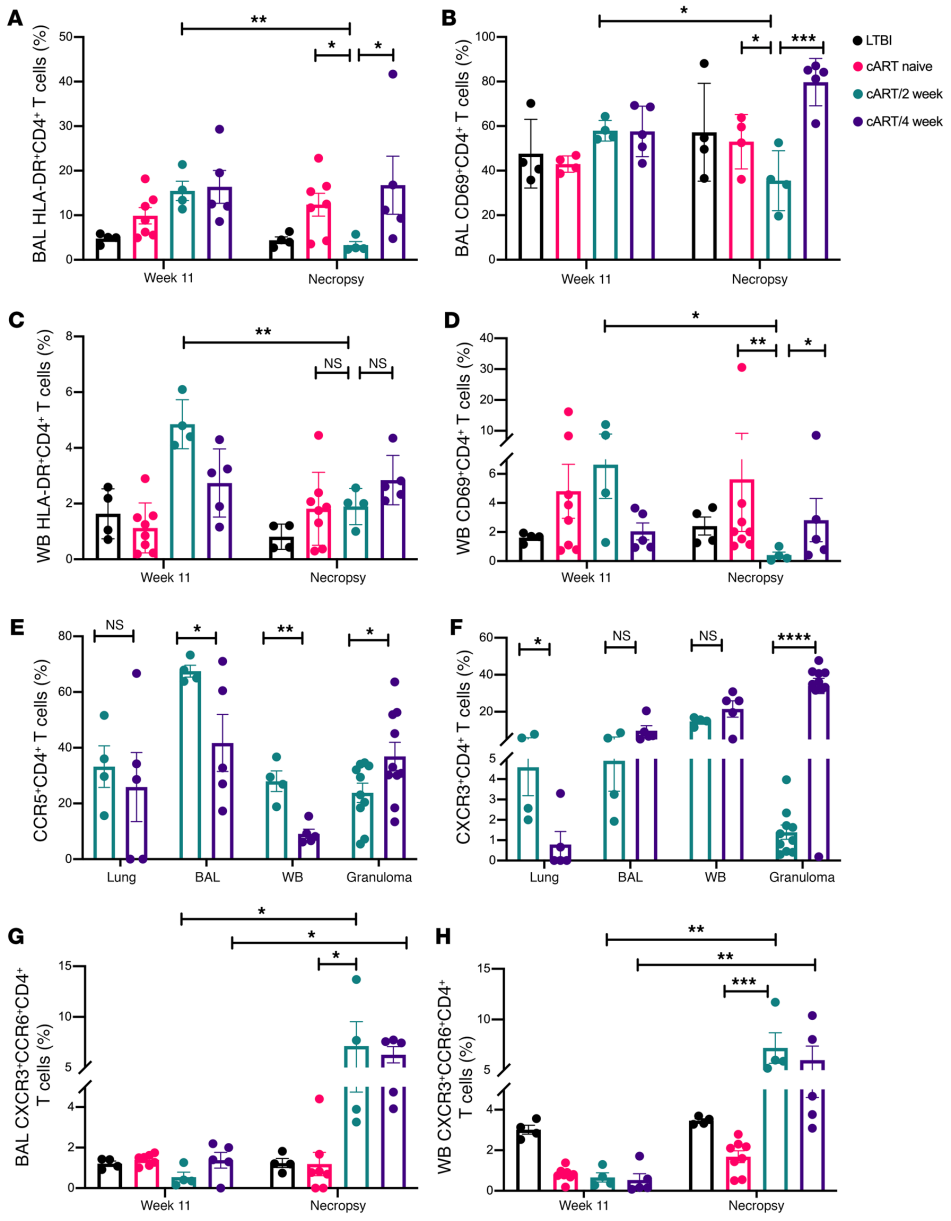
cART initiated at peak viremia better controls immune activation. To study the impact of cART timing on immune activation, we examined the percentages of (**A**) BAL HLA-DR^+^, (**B**) BAL CD69^+^, (**C**) whole-blood HLA-DR^+^, and (**D**) whole-blood CD69^+^CD4^+^ T cells at peak viremia (week 11 after TB) and at necropsy. (**E**) CCR5^+^CD4^+^ and (**F**) CXCR3^+^CD4^+^ T cells were examined in the lungs, BAL, whole blood, and granulomas of cART/2 week (*n* = 4) and cART/4 week (*n* = 5) at necropsy. Percentages of CXCR3^+^CCR6^+^CD4^+^ T cells were examined in the (**G**) BAL and (**H**) whole blood at peak viremia (week 11 after TB) and at necropsy in LTBI (*n* = 4), cART naive (*n* = 8), cART/2 week (*n* = 4), and cART/4 week (*n* = 5). WB, whole blood. Significance was determined using 1-way ANOVA with Tukey’s correction. *P < 0.05; **P < 0.01; ***P < 0.001; ****P < 0.0001. Data are presented as mean ± SEM.

In our previous study, cART was initiated 4 weeks after SIV (13 weeks after *Mtb*; ref. 15). This intervention fails to control chronic immune activation. To study the impact of cART timing on immune activation, we examined HLA-DR^+^CD4^+^, CD69^+^CD4^+^, and PD-1^+^CD4^+^ T cells in BAL and whole blood at peak viremia (week 11 after *Mtb*) and at necropsy in the 4 experimental groups (Figure 5). Early initiation of cART at peak viremia significantly reduced the activation markers, HLA-DR^+^ (*P* = 0.0019) and CD69^+^CD4^+^ T cells (*P* = 0.01), in both BAL (Figure 5, A and B) and the periphery (Figure 5, C and D). A comparable decrease was not observed in the macaques that initiated cART 4 weeks after SIV coinfection (Figure 5, A–D). No significant difference was observed in the immune exhaustion marker, PD-1, in BAL and the periphery between the peak of viral replication and necropsy in any of the 4 experimental groups (Supplemental Figure 4, A and B). Activation markers were also examined in the lungs, BAL, periphery, and granulomas of the 2 treatment groups (Figure 5). A significantly higher (*P* = 0.001) percentage of CCR5^+^CD4^+^ T cells was observed in the BAL and periphery of macaques in the cART/2-week compared with the cART/4-week group (Figure 5E) at study endpoint. Further, markers associated with specific cytokine function of CD4^+^ T cells, CXCR3 (Th1) and CCR6 (Th17), were examined in the tissues and periphery of the treatment groups. As expected, cART treatment resulted in a reversal of the SIV-induced decrease in CXCR3^+^CD4^+^ T cells in the periphery, indicative of viral control (Figure 5F), with no significant differences between the 2 groups. Additionally, a significantly higher (*P* < 0.0001) percentage of Th17 responses (CCR6^+^CD4^+^ T cells) was observed in the granulomas of the cART/2-week group (Supplemental Figure 4C). Concordant with our findings in BAL and the periphery, there was a significantly reduced immune activation and immune exhaustion in lungs and granulomas of the cART/2-week group compared with the cART/4-week group (Supplemental Figure 4, D–F). In conclusion, initiation of cART at peak viremia better controls immune activation and thus LTBI reactivation, though the long-lasting impact will need to be studied after cART termination in longer-tenure NHP studies.

### Early cART initiation fails to reduce inflammation.

To investigate the impact of initiating cART on inflammation, we examined the percentage of CXCR3^+^CCR6^+^CD4^+^ T cells in BAL (Figure 5G) and the periphery (Figure 5H) of macaques in all 4 experimental groups at peak viremia (week 11 after *Mtb*) and at necropsy. A significantly higher (*P* = 0.0006) percentage of CXCR3^+^CCR6^+^CD4^+^ T cells was observed in the BAL and periphery of both the treatment groups compared with LTBI and cART-naive at study endpoint. Despite a decrease in activation markers HLA-DR and CD69 in the cART/2-week group (Figure 5, A and B), the early timing of cART initiation failed to diminish inflammation, both locally and in the periphery (Figure 5, G and H). These findings are in concordance with our earlier studies (15) and with findings in humans (44).

### Early cART reduces macrophage turnover in Mtb/SIV coinfection.

**Figure 6:**
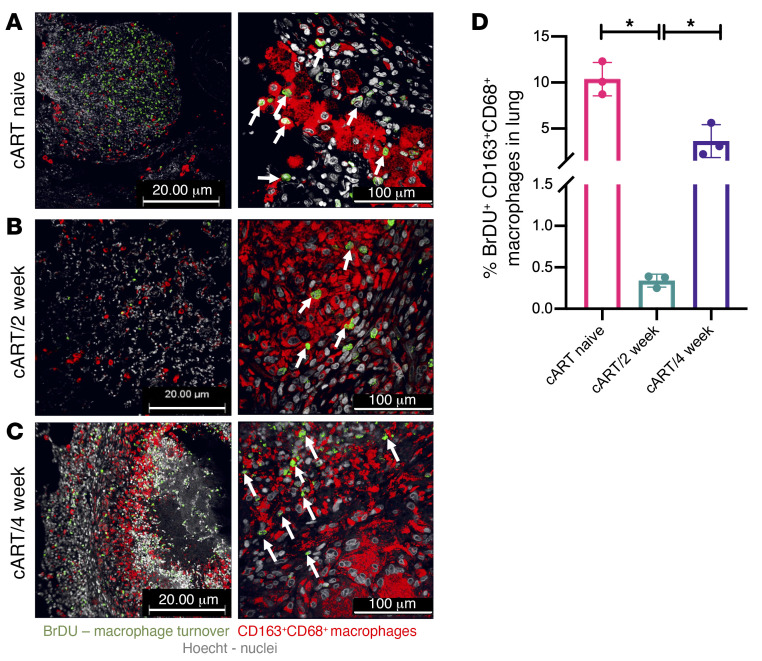
Early cART reduces macrophage turnover in *Mtb*/SIV coinfection. Immunohistochemistry was performed to study the impact of the timing of cART on macrophage turnover by staining for BrDU^+^ nuclei (green, indicated with white arrows) of macrophages (CD163^+^CD68^+^, red) per μm^2^ of lung sections of (**A**) cART naive (*n* = 3), (**B**) cART/2 week (*n* = 3), and (**C**) cART/4 week (*n* = 3) at necropsy. The images were captured on an Axio Scan Z1 and analyzed using HALO software. Significance was determined using 2-tailed Student’s *t* test. *P < 0.05. Data are presented as mean ± SEM. Scale bars: 20 μm (left) and 100 μm (right).

Immunohistochemistry was performed to study the impact of the timing of cART on macrophage proliferation by staining BrDU^+^CD163^+^CD68^+^ macrophages in the lungs of the cART-naive (Figure 6A), cART/2-week (Figure 6B), and cART/4-week groups (Figure 6C). A significantly lower (*P* < 0.05) percentage of macrophage turnover was observed in the lungs of macaques in the cART/2-week group (Figure 6, B and D) compared with the macaques in the cART/4-week group (Figure 6, C and D) and cART-naive group (Figure 6, A and D). The presence of BrDU^+^ nuclei (green) within macrophages (red), as indicated by the white arrows, was observed in the lungs of macaques that received cART 4 weeks after SIV (Figure 6C). This phenomenon was considerably reduced in the macrophages in the lungs of the cART/2-week group (Figure 6B).

### Early cART initiation reduced IDO-1 production in the granulomatous region.

**Figure 7:**
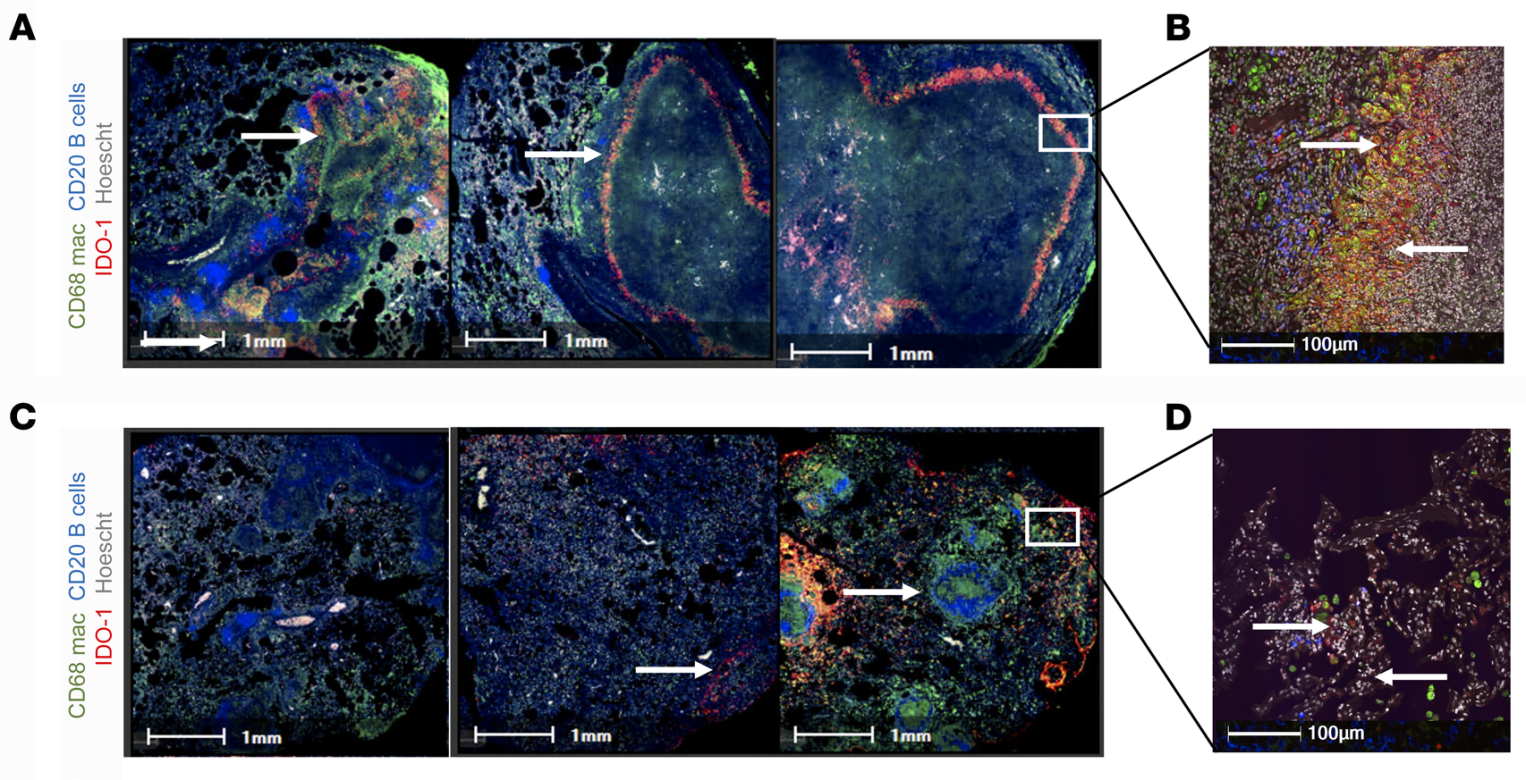
Early cART initiation reduced IDO-1 production in the granulomatous region. Immunohistochemistry was performed to study the impact of the timing of cART on IDO-1 production in the lung tissue by staining for IDO-1^+^ cells (red), macrophages (CD68^+^ green), and B cells (CD20^+^ blue). Panels **A** and **B** represent study animals from cART/4 week (*n* = 5), while panels **C** and **D** represent study animals from cART/2 week (*n* = 4) at necropsy. The arrows point to the IDO-1 production in the granulomatous region and in the lung tissue. The images were captured on an Axio Scan Z1 and analyzed using HALO software. Scale bars: 1 mm (**A** and **C**) and 100 μm (**B** and **D**).

We have previously shown that macrophages expressing indoleamine 2,3-dioxygenase (IDO-1) in the macaque model of *Mtb* infection abrogates CD4^+^ T cell and *Mtb*-antigen-presenting cell interactions (15, 45). In addition, increased bacterial burden and poor formation of inducible bronchus-associated lymphoid tissue (iBALT) correlates with higher expression of IDO-1 in lung tissue (45). Initiating cART 4 weeks after SIV resulted in an increased IDO-1 expression in macrophages surrounding the granulomas with poor iBALT formation (Figure 7, A and B). Earlier initiation of cART drastically reduced the IDO-1 production in the lung tissue (Figure 7, C and D) that corresponds to lower bacterial burden (Figure 2B), improved lung pathology (Supplemental Figure 1C), reduced IFN-γ and TNF-α production (Supplemental Figure 5, A and B), and increased protective IL-17 levels in BAL supernatant (Supplemental Figure 5C).

## Discussion

This is the first study to our knowledge to examine the impact of timing of cART on LTBI reactivation in a biologically and physiologically relevant NHP model. Initiation of cART in HIV-infected individuals has not been standardized with limited clinical trial data to determine the initiation of cART in adults (46–48). Compared with humans who likely develop LTBI with a substantially lower infectious dose of *Mtb* (1–2 CFU), we infected the macaques with approximately 10 to 15 CFU *Mtb* CDC1551. While rhesus macaques infected with this dose/strain combination exhibit control of infection akin to human LTBI, the presented dose is clearly higher than the physiologically relevant human infectious dose. That this results in approximately 70% reactivation in the absence of treatment is therefore expected. Hence, our results are indicative of the worst outcomes in coinfected humans. cART remains the cornerstone of HIV care, though our previous study demonstrated that while cART substantially reduced viral loads, it did not reduce the relative risk of SIV-induced TB reactivation (15). The long-term impact of cART is dependent on the degree of immunodeficiency at which it is initiated (49, 50). In this study, we sought to determine whether initiating cART 2 weeks earlier than the previous study (15), at the time of peak viremia, would better control immune activation and prevent LTBI reactivation. Earlier initiation of cART substantially increased the survival rate of the study macaques with reduced disease severity. In addition, it significantly reduced bacterial burden in lungs and granulomas with no subsequent extrapulmonary spread of the bacteria in spleen or liver. In concordance with these findings, we also observed an improved lung pathology with smaller, rare granulomas and reduced percentage lung involvement in the macaques in cART/2-week group compared with cART-naive controls and the cART/4-week group. Hence, in our model, an earlier initiation of cART resulted in decreased mortality, less disease severity, and improved survival.

Poor CD4^+^ T cell recovery is often associated with persistent immune activation and inflammation (51, 52). We hypothesized that the improved survival could be attributed to an enhanced CD4^+^ T cell recovery in the macaques initiated on cART earlier. In the present study, initiating cART as early as 2 weeks after SIV coinfection restored the lung CD4^+^ T cells to substantially higher levels compared with the cART/4-week group. A better CD4^+^ T cell recovery could be responsible for the improved well being and longevity of the macaques initiated on cART/2-week coinfection. These results reflect human data, wherein earlier initiation of cART in individuals with reduced depletion of CD4^+^ T cells results in limiting chronic immune activation (53). We hypothesize that while earlier initiation of cART is able to restore the CD4^+^ T cell responses better in BAL, the residual chronic immune activation after cART treatment in *Mtb*/SIV-coinfected macaques interferes with the complete restoration of CD4^+^ T cell responses in the lung compartment as well as extrapulmonary organs. Overall, while the timing of cART positively impacts CD4^+^ T cell response restoration, HIV suppression results in better control of CD8^+^ T cell responses in the primary infection site as well as in extrapulmonary organs.

We next determined the phenotype of the replenished CD4^+^ T cells in the lungs of macaques initiated earlier on cART. For this, we characterized the CD4^+^ T cells in the BAL (surrogate for lung) into effector (CD28^–^CD95^+^) and central (CD28^+^CD95^+^) memory phenotypes longitudinally throughout the study. cART initiated as early as 2 weeks after SIV failed to restore the effector memory responses in the BAL to the levels maintained in LTBI. On the other hand, the central memory responses were restored to levels higher or similar to those in the LTBI phase. Further studies should characterize this affected effector phenotype as Th1, Th2, or a mix of Th1 and Th2 to identify the biomarkers and design therapeutics in conjunction with cART. Similar to BAL, we observed a marked increase in the central memory responses in both CD4^+^ and CD8^+^ T cells in the periphery at the end of the study period compared with the effector responses. Thus, cART is unable to restore the CD4^+^ T effector response in the BAL that results in the failure to restore the bacterial control, leading to reactivation despite adequate CD8^+^ T cell responses. We next examined the *Mtb*-specific central and effector memory responses in the primary site of infection, lungs, BAL, and in the extrapulmonary compartments such as bronchial lymph nodes and blood of the macaques initiated on cART 2 weeks after SIV coinfection. Not surprisingly, the fraction of *Mtb*-specific CD4^+^ T effector memory cells was approximately 10%, while the *Mtb*-specific CD4^+^ T central memory cells were at more than 60% in the lungs and BAL. There was no substantial difference in the *Mtb*-specific CD4^+^ T cells producing IFN-γ in the cART/2-week group at necropsy, indicating that while cART has an impact on the *Mtb*-specific effector memory phenotype, there is no marked impact on the production of IFN-γ. It appears that there are factors other than Th1-induced IFN-γ responses that play a role in the control of *Mtb* upon SIV infection and cART administration.

Paradoxically, studies using the macaque model of *Mtb*/SIV coinfection have revealed protective CD4^+^ T cell–independent immune responses that suppress LTBI reactivation (9, 10). Recent work shows that the mere depletion of CD4^+^ T cells is insufficient to cause LTBI reactivation in SIV-coinfected macaques. Instead, chronic immune activation appears to be critical for reactivation (10). Hence, we studied the impact of the timing of cART on the immune activation in the study macaques. An important marker of immune activation following *Mtb*/HIV coinfection in humans is elevated frequencies of HLA-DR^+^CD4^+^ T cells, reflecting chronic T cell activation (54, 55). In the macaques initiated on cART/2 week, there was a significant decrease in HLADR^+^CD4^+^ and CD69^+^CD4^+^ T cells in BAL and in the lungs, from week 11 to necropsy. This was not observed in macaques initiated on cART 4 weeks after SIV coinfection. Early initiation of cART controlled immune activation in BAL to a better extent than the periphery, which is concordant with reduced disease pathology in this group. However, long-term prospective studies are still needed to determine whether early cART translates to a marked reduction in serious non-AIDS events and mortality. CCR5^+^CD4^+^ T cells, a subset of memory/activated CD4^+^ T cells, are both a preferential target of virus replication and a marker of immune activation (56). We observed a substantial number of CCR5^+^CD4^+^ T cells at the end of the study that correlates to the viral replication control by cART and subsequent restoration of CCR5^+^CD4^+^ T cells in all the tissues except in bronchial lymph nodes. We also assessed the markers that are associated with specific cytokine function of CD4^+^ T cells: CXCR3 (Th1) and CCR6 (Th17). In peripheral blood, SIV infection lowers the frequencies of total CXCR3^+^ (Th1) and CCR6^+^ (Th17) T cells. In the coinfected cohort of macaques that were treated with cART at peak viremia, we observed a reversal of the decrease in CXCR3^+^CD4^+^ T cells, with higher levels in the periphery than in the tissue at necropsy, indicative of viral control. In untreated HIV infections, CCR6^+^CD4^+^ T cells are targets of HIV and SIV replication (57). A higher percentage of this subset in the cART/2-week group is indicative of better viral control in the lungs and granulomas. This highlights the need to study the impact of timing of cART on viral reservoirs.

The CXCR3^+^CCR6^+^CD4^+^ subset referred to as Th1* appears to play a critical role in mycobacterial infections in humans (29, 58). A significantly higher percentage of this subset was observed in both the treated groups compared with LTBI and cART-naive groups. However, in people coinfected with pulmonary *Mtb* and HIV, IRIS occurs upon initiation of cART. This TB-IRIS state is characterized by a preferential expansion of CXCR3^+^ and CCR6^+^ populations, as observed in this study. Earlier studies have shown that SIV-driven blood monocyte turnover concurrent with macrophage death is a better correlate of LTBI reactivation than CD4^+^ T cell depletion (8). As in humans, SIV differentially impacts alveolar macrophages (in BAL) and interstitial macrophages in lungs (15). We observed that an earlier initiation of cART at 2 weeks after SIV coinfection in LTBI macaques resulted in a reduced macrophage turnover concurrent with reduced disease pathology. Dissecting the impact of timing and duration of cART on macrophages in lung vasculature is critical to identifying key subsets involved in immune activation and immunosenescence in *Mtb*/SIV coinfection. One of the limitations is that the cART/4-week study was performed prior to the cART/2-week study. Future studies need to be performed to correlate the rate of macrophage turnover to ensuing skewed CD4^+^ T effector functions in the lung and BAL of cART-treated *Mtb*/SIV-coinfected macaques.

In conclusion, initiating cART as early as 2 weeks after SIV coinfection substantially reduced SIV-driven LTBI reactivation by improving the survival and health of the macaques. It effectively controlled SIV replication, improved lung pathology, and reduced T cell activation in the primary site of infection and periphery while maintaining CD8^+^ T effector memory responses. However, it only partially restored CD4^+^ T cells in the lungs and did not rescue from the skewed CD4^+^ T effector memory responses. Earlier initiation of cART could arrest the increase in size of older TB lesions but could not prevent new TB lesions. It failed to contain the virus-induced inflammatory responses in lungs and bronchial lymph nodes, highlighting the role of SIV reservoirs. We hypothesize that future studies on coadministration of anti-TB therapy, such as the WHO-recommended 3HP treatment (once-weekly isoniazid and rifapentine for 12 weeks) of LTBI, with cART could result in enhancement of *Mtb*-specific immunity and prevention of LTBI reactivation compared with cART alone.

## Methods

### Animal infection.

This study included a total of 21 Indian-origin rhesus macaques (*Macaca mulatta*) from 2 different studies. Data were included from completed studies, wherein 17 animals had been enrolled from a specific pathogen–free colony maintained at the Tulane National Primate Research Center (10, 15) and a total of 4 specific pathogen–free Indian-origin rhesus macaques were enrolled from the Southwest National Primate Research Center colony. All macaques were infected with a low dose of approximately 10 CFU *Mtb* CDC1551 (BEI Resources, catalog NR13649) via aerosol as described previously (7, 43, 59, 60). A tuberculin skin test was performed at weeks 3 and 5 after *Mtb* infection to confirm infection. All the macaques were monitored for CRP, percentage body weight, and body temperature weekly throughout the study period. Seventeen of the LTBI macaques were then coinfected with 300 TCID_50_ SIVmac_239_ via the intravenous route 9 weeks after *Mtb* infection (9, 10, 15) (provided by Preston Marx’s Laboratory, Tulane National Primate Research Center). All the procedures were conducted by a board-certified veterinary clinician. The remaining 4 macaques served as LTBI controls for the study. The viral infection was confirmed through plasma viral loads via RT-qPCR. Upon confirmation of SIV infection, the 17 macaques were then divided into 3 groups: the first group of 8 macaques served as coinfected controls with no cART administration, the second group of 4 macaques were started on cART at 2 weeks after SIV coinfection or 11 weeks after *Mtb* infection (cART at peak viremia), and the third group of 5 macaques started cART at 4 weeks after SIV coinfection or 13 weeks after *Mtb* infection (cART at chronic phase of the virus). All the macaques in the ART-at-chronic-phase group had to be euthanized within 9 to 11 weeks of cART treatment (by week 24) due to clinical signs of TB reactivation. The macaques in the cART-at-peak-viremia group were euthanized after 9 weeks of cART treatment to match the treatment tenure with the cART-at-chronic-phase group. The study demographics and the statistical comparison between the 2 studies are presented in Supplemental Tables 1 and 2.

### cART regimen.

Coinfected NHPs received a drug regimen consisting of 20 mg/kg of (*R*)-9-(2-phosphonylmethoxypropyl) adenine (PMPA, tenofovir, Gilead Sciences), 30 mg/kg of 2′,3′-dideoxy-5-fluoro-3′-thiacytidine (FTC, emtricitabine, Gilead Sciences), and 2.5 mg/mL of the integrase inhibitor DTG (ViiV Healthcare). The drugs were administered daily via subcutaneous injection of a cocktail of these 3 drugs in the vehicle KLEPTOSE (Roquette, parenteral grade 346111) at previously published doses (15).

### Viral load and bacterial burden measurement.

Bacterial burden in BAL was measured throughout the study period as previously described (9). Viable *Mtb* burden was also measured at necropsy in BAL, lungs, spleens, bronchial lymph nodes, and individual granulomas collected at necropsy (Figure 2, A–E, and refs. 9, 10). Viral loads in acellular BAL supernatant and plasma were determined by RT-qPCR at peak viremia (2 weeks after SIV or 11 weeks after *Mtb* infection) and at necropsy (Figure 2, F and G). The measurements were performed by the Nonhuman Primate Core Virology Laboratory for AIDS Vaccine Research and Development (Division of AIDS, National Institute of Allergy and Infectious Diseases [NIAID]). A lower limit of 100 copies/sample was set for quantification of SIV copies in this assay.

### CT imaging.

The macaques were anesthetized (Telazol, 2–6 mg/kg; Zoetis) and intubated to perform end-respiratory breath-hold using a remote breath-hold switch. The anesthesia was maintained during imaging by inhalation of isoflurane delivered through the Hallowell 2002 ventilator anesthesia system. Lung field CT images were acquired using a Multiscan LFER150 PET/CT (MEDISO) scanner. 3D ROI Tools available in Vivoquant (Invicro) was used for image analysis. The ventral lung lobes were described as caudal and the upper lung lobes were described as cranial. The CT resolution was fair, with moderate beam hardening/streak artifacts due to cone beam technology. Axial/transverse reconstruction series were provided in soft tissue windows. The studies were reviewed using Sectra IDS7 viewing software in a lung window with centerline –230.0 and window of 2250.0. The CT disease was graded subjectively but utilized the following scheme for image interpretation and grading of mild, moderate, or severe disease: 0, normal; 1, mild interstitial, ground glass opacity or nodules smaller than 5 mm; 2, moderate interstitial, dense ground glass opacity with crazy waving pattern or nodules 5 to 10 mm; 3, alveolar (uniform soft tissue, unable to see vessels) or nodules larger than 1 cm. There was some subjectivity in distinguishing mild and moderate interstitial, and utilizing Hounsfield unit may have been beneficial; however, it was not measured in these studies. If the attenuation in the lung was not uniform and pulmonary vascular margins could readily be delineated, it was graded as mild interstitial/ground glass. If the attenuation in the lung was increased and vascular margins were indistinct or not defined but the attenuation was not uniform soft tissue attenuating, it was classified as moderate. If the pulmonary attenuation was uniform soft tissue with adjacent soft tissue structures, it was classified as alveolar. Pulmonary nodules were graded based on their size: mild, smaller than 5 mm; moderate, 5 to 10 mm; and severe, larger than 1 cm in diameter.

### High-parameter flow cytometry.

High-parameter flow cytometry was performed on BAL cells and whole blood before *Mtb*, before SIV (weeks 3 and 9), after SIV, before cART (week 11), and after cART (week 20 or necropsy). Lungs, bronchial lymph nodes, and granulomas were harvested at necropsy and processed as described previously (9, 10). The prepared single cells were then stained for surface and intracellular markers to study various cell phenotypes (Supplemental Table 3). The freshly collected BAL cells and PBMCs were stimulated ex vivo with *Mtb*-specific antigens, ESAT-6/CFP-10 and *Mtb* Cell Wall Fraction (BEI Resources, 10 μg/mL), for a total of 16 hours. Brefeldin A (0.5 μg/mL, Sigma-Aldrich) was added 2 hours after the onset of stimulation. After stimulation, the cells were stained with LIVE/DEAD fixable Near-IR stain (Thermo Fisher Scientific) and stained subsequently with the following antibodies against cell-surface proteins: anti-CD4–PerCP-Cy5.5 (BD Biosciences, clone L200, catalog 552838), anti-CD8–APC (BD Biosciences, clone RPA-T8, catalog 555369), anti-CD3–Alexa Flour 700 (BD Biosciences, clone SP34-2, catalog 557917), anti-CD95–BV421 (BD Biosciences, clone DX2, catalog 562616), anti-CD28–PECy7 (BD Biosciences, clone CD28.2, catalog 560684), and anti-CD45–BUV395 (BD Biosciences, clone D058-1283, catalog 564099). Cells were then fixed, permeabilized, and stained with the following antibodies against intracellular proteins: anti–IFN-γ–APC-Cy7 (BioLegend, clone B27, catalog 506524), anti–IL-17–BV605 (BioLegend, clone BL168, catalog 512326), and anti–TNF-α–BV650 (BioLegend, clone Mab11, catalog 502938). Cells were washed, suspended in BD stabilizing fixative buffer, and acquired on a BD FACSymphony flow cytometer. Analysis was performed using FlowJo (v10.6.1) software and a previously published gating strategy (Supplemental Figures 6 and 7 and refs. 9, 15, 43).

### Gross pathology.

The macaques were anesthetized for necropsy and lung lobes, spleens, livers, bronchial lymph nodes, BAL, and blood were collected. All the tissues were weighed at the time of collection. Tissues were fixed in 10% neutral buffered formalin, paraffin embedded, sectioned at 5 μm thickness, and stained with H&E using standard methods. Stereology scores were prepared based on percentage lung affected by a board-certified veterinary pathologist.

### Immunohistochemical staining.

Fluorescent immunohistochemistry was performed on formalin-fixed, paraffin-embedded lung and bronchial lymph node tissues as previously described (10, 15, 42, 61). The stained slides were scanned in a Zeiss Axio Scan Z1 and the images were analyzed using HALO software (Indica Labs).

### Statistics.

Statistical analysis was performed using an unpaired Student’s *t* test or 1-way ANOVA with Sidak’s or Tukey’s correction as applicable in GraphPad Prism (version 8.4.1). A *P* value of less than 0.05 was considered statistically significant: *P < 0.05, **P < 0.01, ***P < 0.001, ****P < 0.0001. Data are presented as mean ± SEM.

### Study approval.

All infected macaques were housed under Animal Biosafety Level 3 facilities at the Southwest National Primate Research Center, where they were treated according the standards recommended by AAALAC International and the NIH *Guide for the Care and Use of Laboratory Animals* (National Academies Press, 2011). The study procedures were approved by the Animal Care and Use Committee of the Texas Biomedical Research Institute.

## Author contributions

RS, DK, SAK, SM, and JR designed the study. RS conducted the sample processing and analyzed the flow data with assistance from SRG, DKS, and ANB. RS, XA, and VS performed and analyzed confocal imaging. MG, CA, JF, AB, RT, BS, and RE assisted with necropsy sampling. RS and DK wrote the manuscript. JC supervised the veterinary work. SRG performed the CT scans and aerosol *Mtb* infections. EJD and VS performed the necropsies and histopathology analysis. VS performed the scanning of the confocal slides and the cell counts on the scanned slides. SHU was the attending veterinarian on the study.

## Supplementary Material

Supplemental data

## Version 1 12/02/2021

In-Press Preview

## Version 2 02/01/2022

Electronic publication
