# Supplementary material for: Antiretroviral therapy timing impacts latent tuberculosis infection reactivation in a Mycobacterium tuberculosis/SIV coinfection model
Source: J Clin Invest. 2022 Feb 1;132(3):e153090. doi: 10.1172/JCI153090 (PMC8803324; doi:10.1172/JCI153090)
Supplement: Supplemental data [file jci-132-153090-s078.pdf]

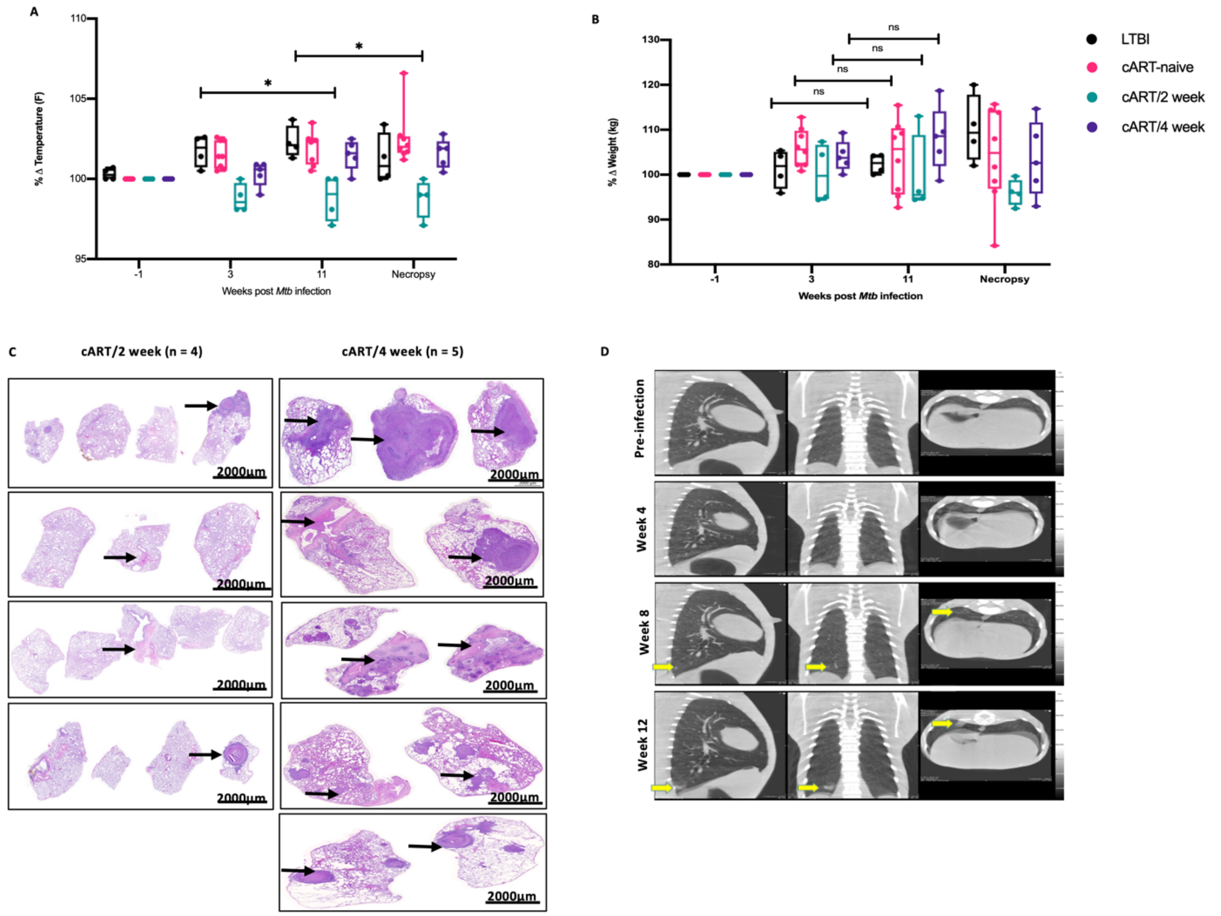

Supplementary Figure 1. (**A**) Percentage temperature change in °F and (**B**) percentage change in body weight (kg) at pre-infection, post-TB infection, post-SIV, pre-cART and post-cART in *Mtb*/SIV co-infected macaques. (**C**) H&E staining of lung sections collected at necropsy from cART treated macaques representing the percent lung involvement. (**D**) Representative CT scan images from cART/2 week (*n*=4) at pre-infection, weeks 4, 8 and 12 post TB infection demonstrating the increase in the size of the older lesions. (**A**) and (**B**) were analyzed using 1-way ANOVA with Tukey's comparison in GraphPad Prism v8.4.1. A *P* value of <0.05 was considered as statistically significant. **P* < 0.05; ***P* <0.01; ****P* < 0.001; *****P* < 0.0001. Data are represented as Mean $\pm$ SEM.

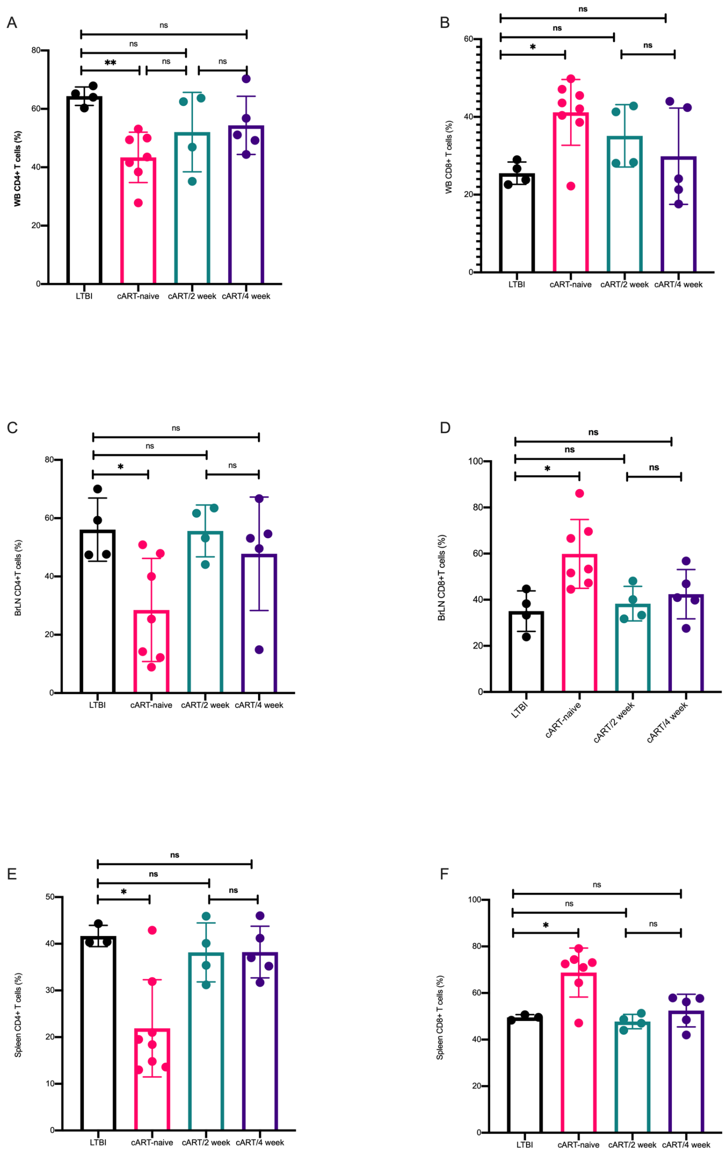

Supplementary Figure 2. $CD4^+$ T and $CD8^+$ T cells enumerated in the (**A, B**) whole blood, (**C, D**) BrLN and (**E, F**) spleen at necropsy in LTBI (*n* = 4), cART naïve (*n* = 8), cART/2 week (*n* = 4) and cART/4 week (*n* = 5). Significance was determined using 1-way ANOVA with Tukey's comparison in GraphPad Prism v8.4.1. A *P* value of <0.05 was considered as statistically significant. **P* < 0.05; ***P* <0.01; ****P* < 0.001; *****P* < 0.0001. Data are represented as Mean ± SEM.

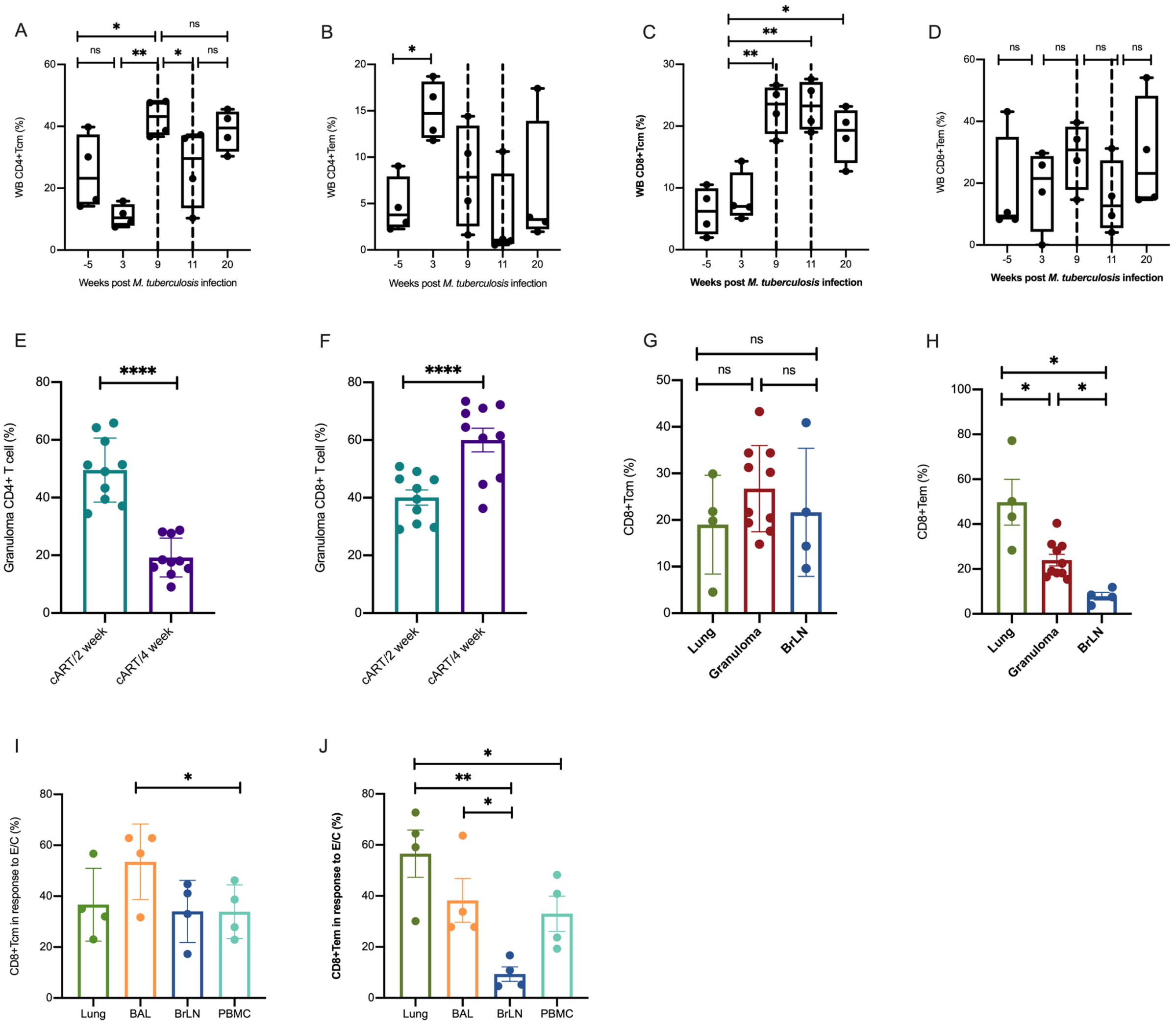

Supplementary Figure 3. Phenotyping of whole blood (A) CD4$^+$ Tcm cells, (**B**) CD4$^+$ Tem cells, (**C**) CD8$^+$ Tcm and (**D**) CD8$^+$ Tem was performed by staining for CD28$^+$CD95$^+$ (Tcm) and CD28$^-$CD95$^+$ (Tem) in cART/2 week (n = 4). Percentage of (**E**) CD4$^+$ T and (**F**) CD8$^+$ T cells in the granulomas of cART/2 week (n = 4) and cART/4 week (n = 5) at necropsy. **(G)** Percentage CD8$^+$ Tcm and (**H**) CD8$^+$ Tem in lung, granulomas and BrLN of cART/2 week (n = 4). Percentage of (**I**) *Mtb*-specific CD8$^+$ Tcm and (**J**) CD8$^+$ Tem responses in lung, BAL, BrLN and PBMCs in cART/2 week (n =4) at necropsy. Significance was determined using 1- way ANOVA with Sidak's or Tukey's correction as applicable in GraphPad Prism v8.4.1. A P value of <0.05 was considered as statistically significant. *P < 0.05; **P <0.01; ***P < 0.001; ****P < 0.0001. Data are represented as Mean $\pm$ SEM.

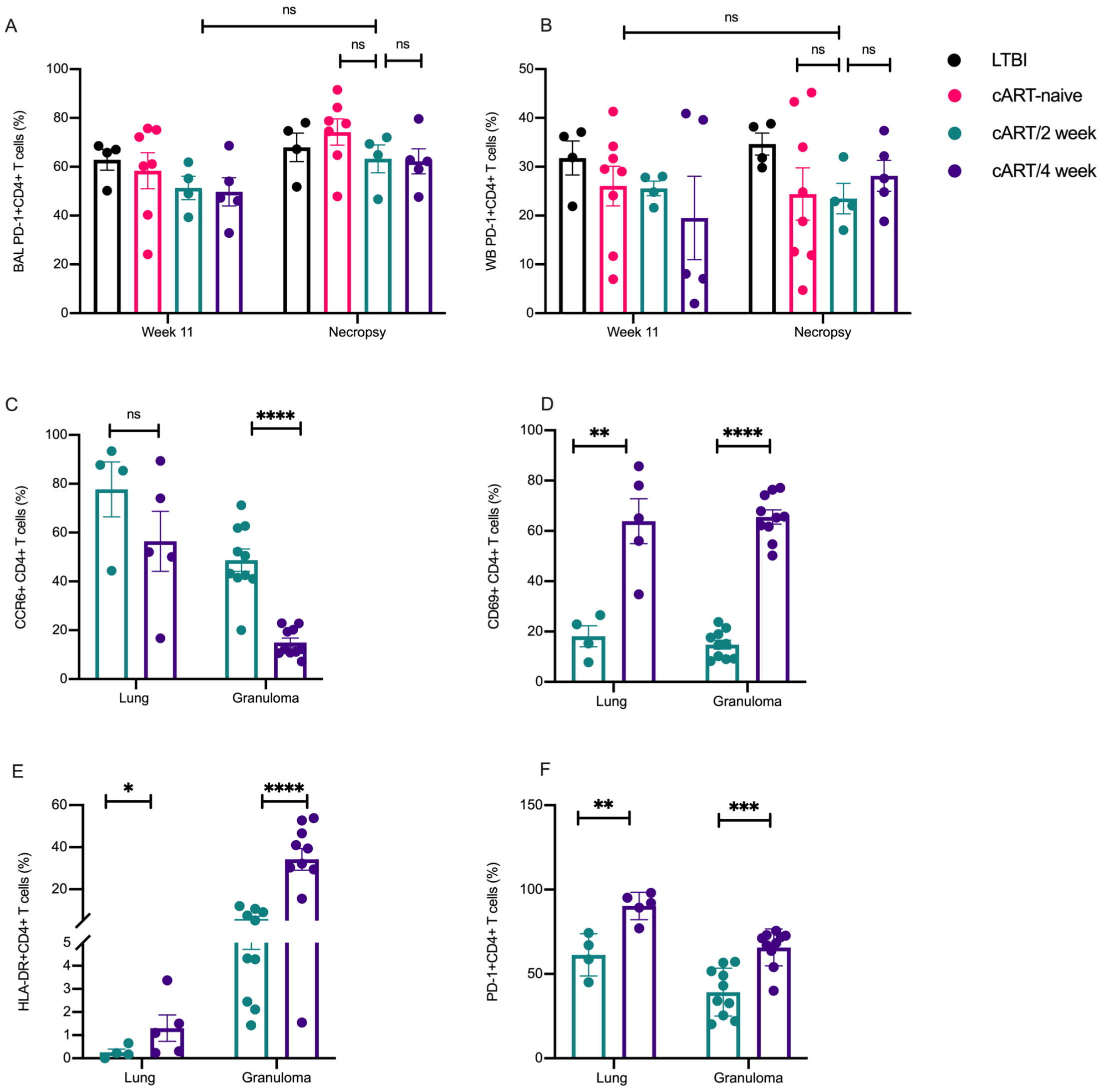

Supplementary Figure 4. Percentage of (**A**) BAL PD-1^+ and (**B**) whole blood PD-1^+CD4^+ T cells in LTBI (n = 4), cART naïve (n = 8), cART 2 weeks post-SIV (n = 4) and cART 4 weeks post-SIV (n = 5) at week 11 post-TB and necropsy time point. Percentage of (**C**) CCR6^+CD4^+ T cells, (**D**) CD69^+CD4^+ T cells, (**E**) HLA-DR$^+$CD4^+ T cells, (**F**) PD-1^+CD4^+ T cells in lungs and granuloma of cART/2 week (n = 4) and cART/4 week (n = 5). Significance was determined using 1-way ANOVA with Tukey's comparison in GraphPad Prism v8.4.1. A P value of <0.05 was considered as statistically significant. *P < 0.05; **P <0.01; ***P < 0.001; ****P < 0.0001. Data are represented as Mean $\pm$ SEM.

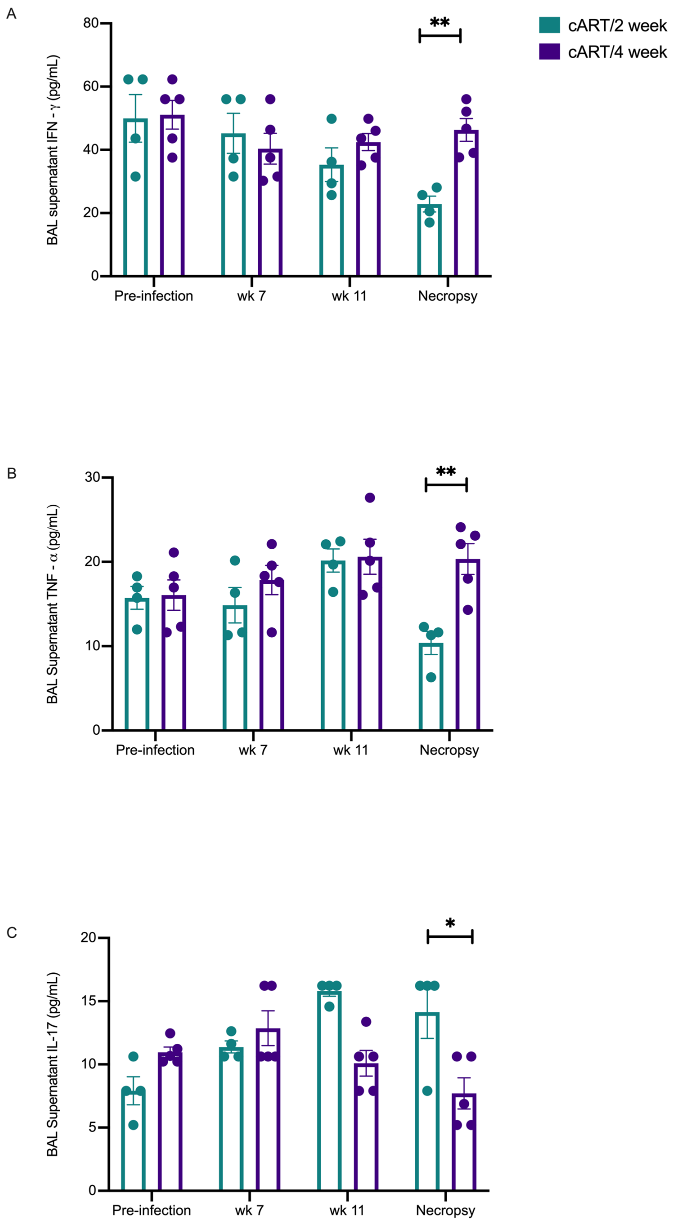

Supplementary Figure 5. Levels of (A) IFN - γ, (B) TNF - α and (C) IL-17 were measured in BAL supernatant at pre-infection, weeks 7, 11 and at necropsy using NHP XL Cytokine Luminex Performance Premixed Kit (R&D systems). The data was analyzed using Belysa software. Significance was determined using 1-way ANOVA with Tukey's comparison in GraphPad Prism v8.4.1. A *P* value of <0.05 was considered as statistically significant. *$P < 0.05$; **$P <0.01$; ***$P < 0.001$; ****$P < 0.0001$. Data are represented as Mean ± SEM.

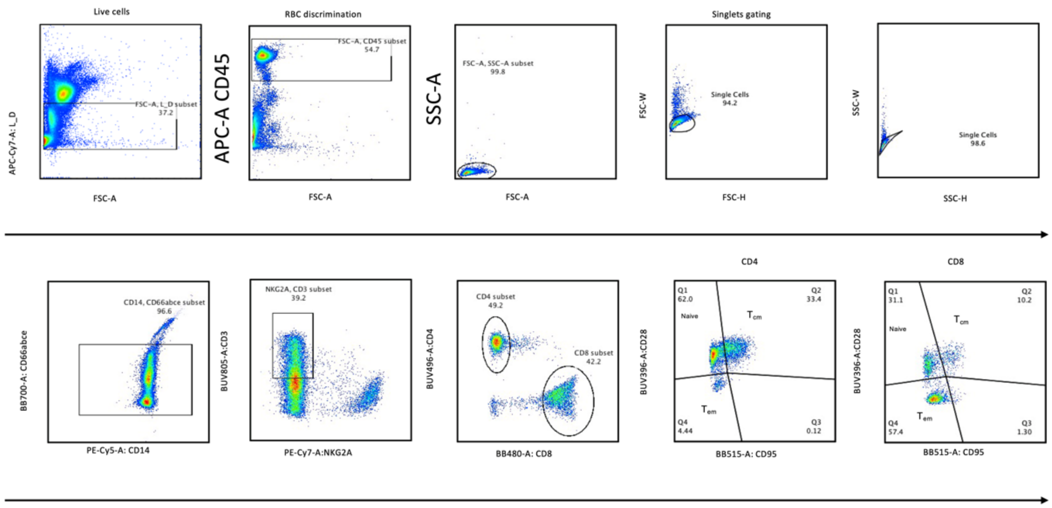

Supplementary Figure 6. Representative gating strategy for CD4 and CD8 T cell memory responses. Lymphocytes were first gated on Live/Dead and CD45 to select for live cells and perform RBC discrimination. This was followed by singlet gating on SSC and FSC -area, width and height. CD66a (CEACAM1), CD66b (CEACAM8), CD66c (CEACAM6), and CD66e (CEACAM5) are differentially expressed on some epithelial cells as well as on T cells, NK cell subsets, and granulocytes. Since our antibody consists of all (CD66a, b, c and e), we gated on total CD66 positive followed by gating out the NK cells and selecting only the total CD3+ population. Next, we gated the CD4 and CD8 on total CD3. Lastly, we gated on CD28 and CD95 for naïve (CD28+CD95-), central memory (CD28+CD95+), effector memory (CD28-CD95+) CD4 and CD8 T cells.

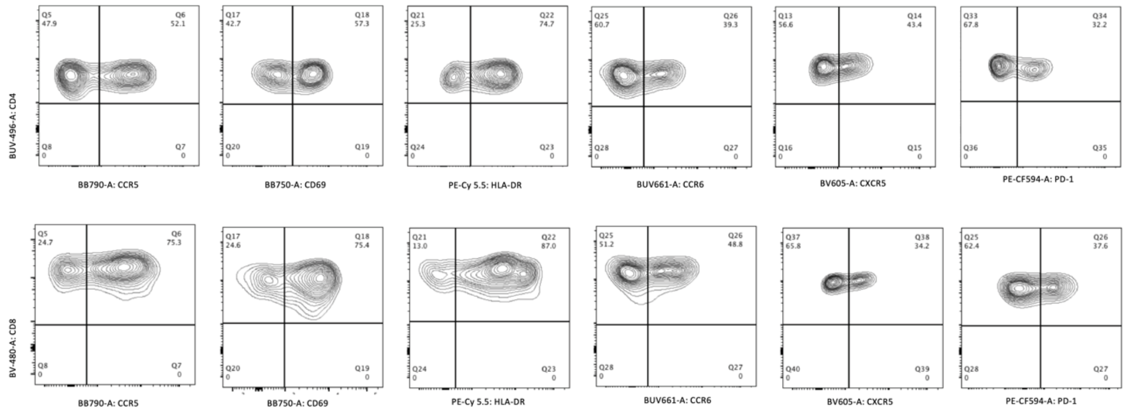

Supplementary Figure 7. Representative gating strategy for CD4 and CD8 T cell transient markers in BAL at week 7 post-*Mtb* infection.

Animal ID	Group	Sex	Age	cART initiation	MAMU Type		
					*A*01*	*B*08*	*B*17*
33343	cART treated	Female	6.30	2 wks post-SIV	Negative	Negative	Positive
33994	cART treated	Male	7.00	2 wks post-SIV	Negative	Negative	Positive
34741	cART treated	Female	5.30	2 wks post-SIV	Negative	Negative	Negative
35974	cART treated	Female	4.40	2 wks post-SIV	Negative	Negative	Positive
32026	cART treated	Male	7.10	4 wks post-SIV	Negative	Negative	Positive
KV48	cART treated	Male	4.82	4 wks post-SIV	Positive	Negative	Negative
LA04	cART treated	Male	4.22	4 wks post-SIV	Negative	Negative	Negative
LA46	cART treated	Male	4.18	4 wks post-SIV	Negative	Negative	Negative
LE24	cART treated	Male	4.03	4 wks post-SIV	Negative	Negative	Negative
KR44	cART naïve	Male	5.50	No cART	Positive	Negative	Negative
LC88	cART naïve	Male	4.10	No cART	Negative	Negative	Negative
JH07	cART naïve	Male	7.32	No cART	Negative	Negative	Negative
JF23	cART naïve	Male	7.26	No cART	Positive	Negative	Negative
KG40	cART naïve	Male	5.51	No cART	Negative	Negative	Negative
IP88	cART naïve	Male	7.05	No cART	Positive	Negative	Negative
JI68	cART naïve	Male	6.06	No cART	Negative	Negative	Negative
JE48	cART naïve	Male	6.33	No cART	Negative	Negative	Negative
GP50	LTBI	Male	11.18	No cART	Positive	Negative	Negative
JF47	LTBI	Male	7.32	No cART	Negative	Negative	Negative
HV02	LTBI	Male	9.34	No cART	Negative	Negative	Negative
JD72	LTBI	Male	7.38	No cART	Negative	Negative	Negative

Supplementary Table 1. Age, sex, timing of cART and Mamu type of the study macaques.

Variable	Study 1*	Study 2**	*P*
N	16	5	
Age (years, mean ± SD)	6.35 ± 2.00	6.02 ± 1.15	0.732
cART Duration (weeks)	6.25 ± 2.5	8.00 ± 2.23	0.302
Sex (N, %)			
Female	0 (0%)	3 (60%)	
Male	17 (100%)	2 (40%)	
Plasma SIV burden at week 11 (peak viremia)	7.07 ± 0.63	7.6 ± 0.30	0.421
BAL Supernatant SIV burden at week 11	4.54 ± 1.52	5.02 ± 0.33	0.552
Mtb burden at week 3 post infection (Log10 CFU/mL)	0.50 ± 0.82	0.34 ± 0.68	0.731

*KV48, LA04, LA46, LE24, KR44, LC88, JH07, JF23, KG40, IP88, JI68, JE48, GP50, JF46, HV02, JD72 (14)

**33343, 33994, 34741, 35974, 32026 (current study)

P values for numerical variables are based on Kruskal-Wallis test across all groups.

For pairwise comparisons, P-values are based on Wilcoxon test, adjusted for multiple comparisons (Holm)

A *P* value of <0.05 was considered as statistically significant.

* *P* < 0.05; ***P* <0.01; ****P* < 0.001; *****P* < 0.0001. Data are represented as Mean ± SD

Supplementary Table 2: Comparison of study demographics

Marker	Fluorochrome	Clone	Catalog No.
CD28	BUV395	CD28.2	740308
CD4	BUV496	L200	750591
CD196(CCR6)	BUV661	11A9	750696
CD3	BUV805	SP34-2	742053
CD8	BV480	RPA-T8	566163
CD183(CXCR3)	BV570	1C6/CXCR3	624298
CD185(CXCR5)	BV605	MU5UBEE	63-9185-42
CD69	BV750	FN50	747522
CD103	BV786	B-Ly7	78-1038-41
CD95	BB515	DX2	564597
CD66abce	PerCP-Cy5.5	TET2	130-119-936
CD195(CCR5)	BB790	3A9	624296
PD-1	PE-CF594	EH12.2H7	329939
HLA-DR	PE-Cy5.5	L243	624294
CD45	APC	D058-1283	561290

Supplementary Table 3. High parameter flow cytometry panel. 9
